# Integrated Transcriptome and Metabolomic Analysis Reveal Anti-Angiogenic Properties of Disarib, a Novel Bcl2-Specific Inhibitor

**DOI:** 10.3390/genes13071208

**Published:** 2022-07-06

**Authors:** Meghana Manjunath, Sai Swaroop, Sai Sanwid Pradhan, Raksha Rao K, Raghunandan Mahadeva, Venketesh Sivaramakrishnan, Bibha Choudhary

**Affiliations:** 1 Institute of Bioinformatics and Applied Biotechnology, Bengaluru 560100, Karnataka, India; mmeghana@ibab.in (M.M.); raksharaok@gmail.com (R.R.K.); raghunandan.hunsur@gmail.com (R.M.); 2 Manipal Academy of Higher Education, Manipal 576104, Karnataka, India; 3 Disease Biology Lab, Department of Biosciences, Sri Sathya Sai Institute of Higher Learning, Anantapur 515001, Andhra Pradesh, India; saiswaroopr@sssihl.edu.in (S.S.); saisanwidpradhan@sssihl.edu.in (S.S.P.); svenketesh@sssihl.edu.in (V.S.)

**Keywords:** transcriptomics, metabolomics, Bcl2 inhibitors, gene expression, LC-MS, oncometabolite, angiogenesis

## Abstract

Transcriptomic profiling of several drugs in cancer cell lines has been utilised to obtain drug-specific signatures and guided combination therapy to combat drug resistance and toxicity. Global metabolomics reflects changes due to altered activity of enzymes, environmental factors, etc. Integrating transcriptomics and metabolomics can provide genotype-phenotype correlation, providing meaningful insights into alterations in gene expression and its outcome to understand differential metabolism and guide therapy. This study uses a multi-omics approach to understand the global gene expression and metabolite changes induced by Disarib, a novel Bcl2-specific inhibitor in the Ehrlich adenocarcinoma (EAC) breast cancer mouse model. RNAseq analysis was performed on EAC mouse tumours treated with Disarib and compared to the controls. The expression of 6 oncogenes and 101 tumour suppressor genes interacting with Bcl2 and Bak were modulated upon Disarib treatment. Cancer hallmark pathways like DNA repair, Cell cycle, angiogenesis, and mitochondrial metabolism were downregulated, and programmed cell death platelet-related pathways were upregulated. Global metabolomic profiling using LC-MS revealed that Oncometabolites like carnitine, oleic acid, glycine, and arginine were elevated in tumour mice compared to normal and were downregulated upon Disarib treatment. Integrated transcriptomic and metabolomic profiles identified arginine metabolism, histidine, and purine metabolism to be altered upon Disarib treatment. Pro-angiogenic metabolites, arginine, palmitic acid, oleic acid, and myristoleic acid were downregulated in Disarib-treated mice. We further validated the effect of Disarib on angiogenesis by qRT-PCR analysis of genes in the VEGF pathway. Disarib treatment led to the downregulation of pro-angiogenic markers. Furthermore, the chorioallantoic membrane assay displayed a reduction in the formation of the number of secondary blood vessels upon Disarib treatment. Disarib reduces tumours by reducing oncometabolite and activating apoptosis and downregulating angiogenesis.

## 1. Introduction

Breast cancer is a heterogeneous disease with a high diversity between and within subtypes. Recent advances in cancer development have led to selective targeting of deregulated molecules and pathways to achieve a rational cancer therapy. Such targets include mutant kinases, cancer stem cells, and tumour microenvironment, etc. [1]. The Bcl2 family of proteins are central regulators of the mitochondrial pathway of apoptosis, and more than half of the cancers show Bcl2 (anti-apoptotic protein) overexpression. BCL2 is overexpressed in ~75% of breast cancer and 41% of triple-negative breast cancers [2,3], thus making it an ideal target for cancer therapy [4]

Many small-molecule inhibitors, such as Gossypol, AT101, Obatoclax, ABT-737, ABT-263, Tw37, YC137, and HA-14 break, and the interaction between Bcl2 and its partner Bak/Bax (pro-apoptotic) has been developed [5,6,7,8,9]. ABT-199 is Bcl2 specific, highly potent inhibitor and has been approved by the FDA for leukaemia and lymphoma [6]. However, resistance to ABT-199 has been observed in patients [7,8,9]. We have synthesised a small molecule inhibitor against Bcl2, Disarib, which exhibited cytotoxicity in Bcl2 ‘high’ cancer cell lines and CLL patient primary cells [10,11]. Disarib disrupts Bcl2-Bak interaction while sparing Bcl2-Bax and is a promising Bcl2 inhibitor [10]. Disarib has also shown an effect in Ehlrich adenocarcinoma and Dalton lymphoma mouse models [11]. Since Disarib targets Bcl2, any cancer with a high Bcl2 level might benefit from Disarib treatment. To understand the changes in global gene expression and the metabolic end-products brought about by Disarib, we tested Disarib on the mouse breast cancer model. Moreover, understanding the complete mechanism of action of these inhibitors is necessary to combat drug resistance.

One of the strategies explored to get insight into the drug-induced changes at the transcriptional level is RNA-Seq. RNA-seq allows deciphering the biological effects of the drug, in vitro, and in vivo [12]. One of the first efforts toward measuring alterations in gene expression resulted in creating the Connectivity Map. Connectivity Map provides the expression of 1000 genes of significance in disease states [13]. Metabolomics is a recent omics technology that utilises modern analytical instrumentation combined with pattern recognition methods to identify metabolic changes in a diseased condition or any other medical intervention [14,15]. Transcriptomics, proteomics, and metabolomics combined have helped unravel the drug resistance mechanisms and repurpose the drugs for chemotherapy. Several applications, such as pathway analysis and toxicity models, have been developed in line with gene expression analysis. Integrated transcriptome and metabolome analysis in obese breast cancer patients revealed alterations in glutathione metabolism, glycine and serine metabolism, valine, leucine, and isoleucine degradation, and elevated purine metabolism [16].

Bcl2 is an antiapoptotic protein which regulates cancer cell survival by altering the pro-apoptotic/antiapoptotic balance in the cell and promotes angiogenesis [17,18]. Angiogenesis is the formation of new blood capillaries from an already existing vasculature. Solid tumours utilise the blood vessels around them to metastasise to different organs for nutrients and remove metabolic wastes [19,20]—Angiogenesis results from a tilt in the balance between pro and anti-angiogenic factors. VEGF and its receptors, VEGFRs are the significant angiogenesis activators [21,22]. Other players angiotensin1,2 and Tie 2 stabilise the newly formed capillaries [23]. One of the Bcl2 inhibitors, Tw37, inhibits angiogenesis in in vivo models [24].

This study employs integrated transcriptomic and metabolomic approaches to study the effect of Disarib in vivo in an EAC-induced breast cancer model.

## 2. Materials and Methods

### 2.1. Chemicals and Reagents

All the chemicals used for the experiments were purchased from Biorad (Hercules, CA, USA), Thermo Fisher Scientific (Waltham, MA, USA), MP biomedicals (Santa Ana, CA, USA), Life Technologies (Carlsbad, CA, USA), KAPA (Basel, Switzerland), Sigma Chemical Co. (St. Louis, MO, USA), and BioLegend (San Diego, CA, USA). Primers were ordered from Bioserve Biotechnologies India Pvt Ltd. (Hyderabad, India). NGS reagents were purchased from Illumina (San Diego, CA, USA) and New England Biolabs (NEB, Ipswich, MA, USA). Analytical quality water used for all the studies was obtained from the MilliQ water purification system from Thermo Fisher Scientific, USA. All other chemicals used were of analytical grade. Disarib, the BCL2-specific inhibitor was synthesized in-house [25].

### 2.2. Animals

Swiss albino mice experiments were conducted per the Institutional Animal Ethical Committee guidelines of IBAB and as per Indian national animal care and use law (Ref. IAEC/IBAB/07/10-7-2019). Female Swiss albino mice of weight 19–22g 4–6 weeks old were purchased from Liveon Biolabs Pvt. Ltd., Bangalore, India. Animals were maintained in a room with a 12 h dark/12 h light cycle and controlled humidity and temperature (23 + 3 °C). Ventilated polypropylene cages were used for the animals, and they were supplied with a standard pellet diet (Liveon Biolabs, India) and water ad libitum. Components of the standard pellet diet is 21% protein, 5% lipids, 4% crude fibre, 8% ash, 2% vitamin, 3.4% glucose, 1% calcium, 0.6% phosphorus, and 55% nitrogen-free extract (carbohydrates).

### 2.3. Ehrlich Ascites Breast Adenocarcinoma (EAC) Tumour Model

**Figure 1:**
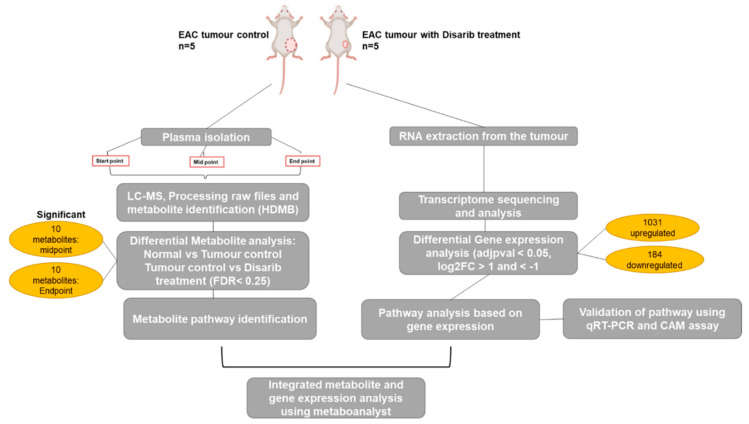
Experimental approach used for the study. Plasma was isolated from the blood of tumour control, Disarib treated and normal mice control, and Disarib treated at three different time points. Tumours from the control and treated animals were used to isolate RNA and perform transcriptome analysis. LC-MS was performed, and a metabolite profile was obtained for each sample. Transcriptome and metabolome data were further integrated, and significant pathways were obtained upon Disarib treatment.

A total of 20 Swiss albino mice were used for the study. Among these, 10 served as no tumour control. Intramuscular injection of EAC cells (1 × 10^6^ cells/animal) into the left thigh was given to 10 animals for developing a solid tumour. The no-tumour control animals were divided into two groups; Group I served as tumour control and received no treatment, and Group II received 50 mg/kg Disarib orally. EAC injected animals were divided into 2 groups containing 5 animals each (Group III and IV). Group III animals were EAC tumour controls that did not receive any treatment, and group IV received 50 mg/kg of Disarib orally. The treatment was given after 7 days of tumour development and continued until the 22nd day (12 doses). The tumour growth was monitored by measuring the diameter of the tumour using vernier callipers every alternate day until a 70–80% reduction of the tumour was observed. The tumour volume was calculated using the formula V = 0.5 × a × b2, where ‘a’ and ‘b’ indicate major and minor diameter, respectively. Blood was collected from the tail vein of the animals belonging to groups I, II, III and IV at three different time points; (a) after tumour development, (b) midway through Disarib treatment, and (c) once the tumour was reduced by around 70–80%, plasma was isolated from the blood samples and subjected to metabolomics analysis [26,27]. Once the tumour was reduced by around 70–80%, it was collected in trizol and stored at −80 until further use for RNA seq analysis (Figure 1).

### 2.4. RNA Extraction and Library Preparation

RNA was isolated using the standard Trizol method [24]. Briefly, to the trizol, 2 M sodium acetate (pH 4) and chloroform were added. The aqueous layer was collected and precipitated by adding an equal volume of isopropanol. Pellet was washed with 80% ethanol, air-dried and resuspended in DEPC-treated Milli-Q water. RNA was quantified using Qubit, and the quality was checked on a tape station. mRNA libraries were prepared using Illumina TruSeq RNA Library Prep Kit v2. Briefly, mRNA was isolated using oligo-dT beads and followed by fragmentation. Fragmented RNA was then converted to cDNA, and adaptor ligation was performed. Size selection was performed on Adaptor ligated libraries using ampure beads. The libraries were then amplified and checked on a tape station to determine the library size [27,28]. A pool of the libraries was prepared and loaded onto the flow cell of Illumina Hiseq 2500 [29,30].

### 2.5. Processing and Alignment of Fastq Files

The samples were sequenced in-house using Illumina Hiseq2500 to acquire 100bp paired-end reads. Samples had reads greater than 10 million. The quality of the reads was checked using the Fastqc tool [28]. The reads were then aligned to the reference mm10 (Downloaded from The University of California, Santa Cruz (UCSC) genome browser) for mouse samples using bowtie2 with default parameters [29]. A SAM (Sequence alignment map) format file was obtained as an output of the bowtie2. A binary alignment map (BAM) file was obtained using Samtools [30] from the SAM file. An annotation file for mouse mm10refseq.bed was downloaded from UCSC for humans, and read counts were generated using bed tools [31].

### 2.6. Normalisation and Differential Gene Expression Analysis

The read counts were quantile-normalised using the R package. Normalised read counts were subjected to differential analysis [32]. A differential gene expression was performed between EAC tumour control and Disarib treated EAC tumour samples. The R package was used to find differential gene expression [33]. RPKM was also used for normalising the data [34]. ‘dist’ in R was used to calculate the Euclidean distance between samples. Normalised read counts were given as an input to Deseq2’s plot PCA function to perform principal component analysis (PCA).

### 2.7. Significant Gene List Analysis

From the EAC tumour treated with Disarib samples, a cutoff of *p*-value adjusted less than 0.05, and log2 fold change (<−1 and >+1) was applied to obtain a significant DEG list. A list of oncogenes and tumour suppressor genes specifically for breast cancer was procured from https://oncovar.org/ and https://bioinfo.uth.edu/TSGene/, accessed on 25 June 2021, respectively. Downregulated oncogenes and upregulated tumour suppressor genes were obtained from the significant gene list of Disarib-treated EAC tumours. Heatmap was plotted to analyse oncogenes and tumour suppressor genes using pheatmap, an R package. String database was used to generate a network of the oncogenes, tumour suppressor genes and BCL2. Significantly up and downregulated genes were separately given as an input to the Reactome database [35] to obtain significantly deregulated pathways upon treatment of Disarib. The results were plotted as a bubble plot using ggplot2, an R package [36].

### 2.8. First-Strand cDNA Synthesis

Complementary DNA was synthesised using NEB reagents from the intact RNA [37,38]. 4 µg of RNA was taken from EAC tumour control and Disarib-treated tumour. To remove DNA contamination, RNA samples were treated with DNase (37 °C, 10 min) before cDNA synthesis. cDNA synthesis reaction mix was prepared using M-MuLV reverse transcriptase, adaptor primers and dNTPs, and Random primers (NEB) (37 °C, 1 h) [39]. A reaction without the RTase enzyme was used as a negative control.

### 2.9. Real-Time PCR

Real-time PCR was conducted using SYBR^®^ Green chemistry. Primers for angiogenesis marker genes were used with GAPDH primer as an internal control (Supplementary Table S1). The initial denaturation was done at 95 °C for 5 min and followed by the cycling stage (40 cycles, 95 °C for 20 s, 53 °C for 20 s, 72 °C for 20 s) and melt curve stage [40,41,42].

### 2.10. Comparative Ct Analysis for Relative Gene Expression Analysis

The relative gene expression was calculated by correlating the housekeeping gene’s expression and the target gene’s expression in the control/normal sample. Ct is the cycle number where the fluorescence crosses the threshold level [43,44]. The relative quantitation (RQ) value equation is

where

ΔΔCt = ΔCt _(Control)_ − ΔCt _(Disarib treated)_

And

ΔCt _(Control)_ = Ct _(target gene of control sample)_ − Ct _(housekeeping gene of control sample)_

ΔCt _(Disarib treated sample)_ = Ct _(target gene of Disarib treated sample)_ − Ct _(housekeeping gene of Disarib treated sample)_ (1)

Graphs showing relative quantification for all the samples were plotted using the GraphPad prism software [45].

### 2.11. Metabolite Analysis through Mass Spectrometry

#### 2.11.1. Sample Preparation

Plasma samples from the animals were mixed with 2.5 µM of labelled Internal Standards Solution (ISTD) made with 50% methanol and incubated in ice for 30 min. Samples were sonicated after the incubation for 15 min. The supernatant was then filtered using an amicon 3kDa cutoff filter. A 2 µL of the supernatant was then analysed in Agilent 6490 iFunnel triple quadrupole LC/MS system.

#### 2.11.2. Solvent Preparation

Waters X-Bridge amide 3.5 µm, 4.6 × 100 mm column was utilised for the study (positive mode). 100% acetonitrile with 0.1% formic acid was solvent-B (organic), and water with 0.1% formic acid was solvent-A (aqueous). The solvents were subjected to sonication for 15 min before use [46,47]. The sample run and analysis were performed as standardised earlier [48,49].

#### 2.11.3. Instrument

This study used a triple-quadrupole mass analyser for the targeted metabolomics. Quadrupole analysers are simple, robust and with good low mass resolution and accuracy [50]. The ions are separated based on flight trajectory stability via an oscillating electric field. The metabolites can be scanned for a range of m/z values by changing voltages continuously [51]. The Agilent 6490 iFunnel triple quadrupole LC/MS system was used to identify metabolites. A Flow rate of 0.3 mL/min in a gradient from 15:85% from 0th to 3rd min, 70:30% from 3rd to 12th min, 98:2% from 12th to 15th min, 98:2% from 15th to 16th min, 15:85% from 16th to 23rd min and 15:85% from 23rd to 28th min of solvent A and solvent B respectively. The Instrument was adjusted to 400 V delta EMV, 3000 V capillary voltage, 250 °C capillary temperature, 350 °C sheath gas heater temperature, 12-unit sheath gas flow, and 20 psi nebuliser pressure (Supplementary File S2). To evaluate the consistency and reproducibility of the analysis, three tubes of pooled serum (quality control) were also extracted and analysed in similar way as to test samples by injecting them at the beginning, middle, and end of the run (Supplementary File S3).

#### 2.11.4. Metabolome Analysis

Agilent MassHunter Qualitative Analysis B.06.00 and Agilent MassHunter Quantitative analysis B.06.00 software (Santa Clara, CA, USA) was used for analysis. The list of metabolites was analysed based on Retention time, area under the peak and signal-to-noise ratio. The selected metabolites were then normalised w.r.t internal standard and subjected to binary logarithmic transformation (base 2). Statistical analysis was performed using Metaboananalyst (Version 5.0) https://www.metaboanalyst.ca/) accessed on 25 June 2021. (An open-source software for analysis) Edmonton, Alberta, Canada. Both univariate and multivariate regression analysis was performed on the metabolites. Univariate analysis (*t*-test) generated metabolites with t-stat and False discovery rate (FDR) value. Significant metabolites (FDR ≤ 0.25) were subjected to pathway analysis, and a heatmap was plotted using the same software for all the time points [52]. Multivariate analysis using partial Least Squares—Discriminant Analysis (PLS-DA) that utilises regression and extracts information through a linear combination of variables to predict classification was also performed. PLS-DA calculates the weighted sum of squares of the loadings and generates Variable Importance in Projection (VIP) scores for each metabolite. VIP scores ≥ 1 were plotted for each time point and were further analysed [53]. Significant DEGs (*p*-value adjusted less than 0.05, and log2 fold change (<−1 and >+1)) and metabolites with VIP scores ≥ 1 were used for generating interaction networks using Metaboanalyst software Edmonton, Alberta, Canada.

#### 2.11.5. Chorioallantoic Membrane (CAM) Assay for Checking Angiogenesis

CAM is an extra-embryonic membrane comprised of a high density of blood and lymphatic vessels [53]. CAM has a dense capillary network formed by the fusion of two mesodermal layers, chorion and allantois and is commonly used to study in vivo angiogenesis and anti-angiogenesis in response to potential biomolecules and drugs [54,55]. A window was made on the 3-day-old fertilised egg. Disc with 100 µM, 250 µM and 500 µM Disarib were placed inside the egg near the blood vessels on day 5. After 48 h, a photograph was taken to observe any changes in the blood vessels. A minimum of 5 eggs was used for control and treatment groups; secondary blood vessels were counted and plotted as a bar graph using GraphPad prism

#### 2.11.6. Statistical Analysis

Statistical analyses and graphing were done using GraphPad Prism 7.0 software (GraphPad, San Diego, CA, USA) and R packages, Vienna, Austria. Deseq2 uses the Wald test statistic with a probability to generate a significant gene list. The Benjamini–Hochberg False Discovery Rate (FDR) method was used for choosing significant pathways from the Reactome database. For comparative qRT-PCR analysis, a two-tailed *t*-test was applied to calculate the significance. *p* values less than 0.05 were considered significant, and the bar graphs are represented as Mean + SEM. *p* < 0.05 = *, *p* < 0.01 = **, *p* < 0.001 = ***, *p* < 0.0001 = **** were used for representation.

## 3. Results

### 3.1. Principal Component Analysis Segregated Control, and Disarib Treated EAC Samples

**Figure 2:**
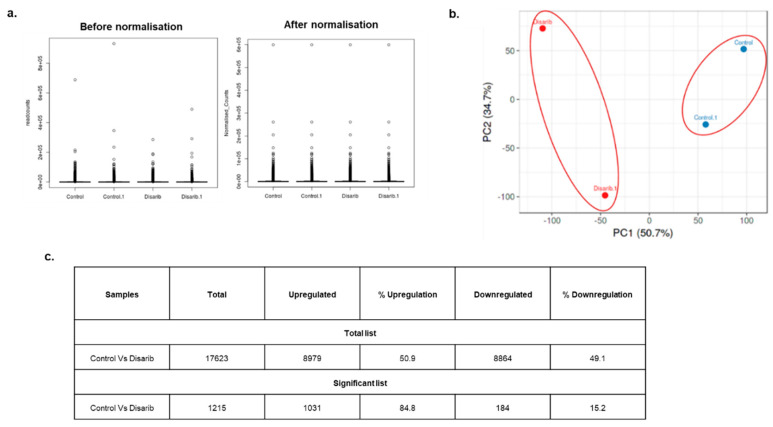
Preliminary analysis of EAC Control and treated samples. (**a**) Quantile normalised plots for EAC control and Disarib treated samples before and after normalisation. (**b**) Principal component analysis of EAC control and treated samples. Blue dots represent control, and red dots represent Disarib treated samples. (**c**) Table depicting the number of up and downregulated genes upon Disarib treatment in EAC tumour samples.

EAC tumour control and Disarib treated tumour RNA samples were sequenced (2 replicates each). Around 50–70 million reads were obtained for all the samples after sequencing (Supplementary Table S2). The quality of the sequences was verified through fastqc, the bad quality bases were trimmed, and the reads were aligned to the mouse reference genome (mm10). The alignment percentage for all the samples was around 75–85%. Quantile normalisation of the read counts was performed to obtain similar distributions for all the samples (Figure 2a). The quantile normalised samples were then subjected to principal component analysis to evaluate the resemblance and differences between the control and treatment groups. Principal Components Analysis reduces the overwhelming number of data dimensions conserving the original information in the data to the maximum. The PCA plots of EAC samples revealed that the control and treated samples clustered separately (Figure 2b). Control and treated samples showed variation in the first dimension suggesting variation between the two groups. However, the replicates are segregated in the second dimension, indicating the similarity.

### 3.2. EAC Samples Displayed Equal Upregulation and Downregulation of Differentially Expressed Genes

The quantile normalised read counts were subjected to differential gene expression analysis using deseq2 (R package). The analysis revealed 17,623 differentially expressed genes between the EAC control and Disarib-treated tumour samples (Supplementary File S1). 8979 (50.9%) were upregulated, and 8864 (49.1%) were downregulated. A sub-list was created from the main DEG list by putting a cutoff of *p*-value-adjusted less than 0.05 and log2fold change greater than 1 and less than −1 to filter out the significant genes. A total of 1215 DEGs were significant, out of which 184 were downregulated, and 1031 were significantly upregulated. There was equal up and downregulation of genes in the total DEG list. However, most of the significant DEGs showed upregulation. The number of DEGs for each sample is summarised in Figure 2c.

### 3.3. Disarib Modulates the Expression of Oncogenes and Tumour Suppressors in EAC Tumour

Significant DEGs from Disarib-treated EAC tumour samples were subjected to oncogenes and tumour suppressor gene analysis. Breast cancer-specific oncogene lists were obtained from the OnGene database, and tumour suppressor genes were procured from the TSgene database. Disarib significantly downregulated 6 oncogenes and upregulated 101 tumour suppressor genes (Supplementary Figure S1a). These genes were then subjected to network analysis using the STRING database. A network was constructed, k means clustering was performed, and the line thickness between the genes indicates the confidence of the interaction. The results suggest that most oncogenes and tumour suppressor genes altered by Disarib interact directly or indirectly with BCL2, BAK and BAX (Supplementary Figure S1b). Csf3r, Cxcl1, Hax1, Ddit3, Pa2g4 and Mnat1 were the oncogenes participating in nucleotide excision repair; basal transcription factors, RNA polymerase II transcription was downregulated upon Disarib Treatment. Tumour suppressor genes participating in ERBB signalling, proteoglycans and apoptosis were upregulated.

### 3.4. Disarib Induced Shrinkage of EAC Tumours Correlated with Downregulation of Pathways in Cancer Hallmarks

**Figure 3:**
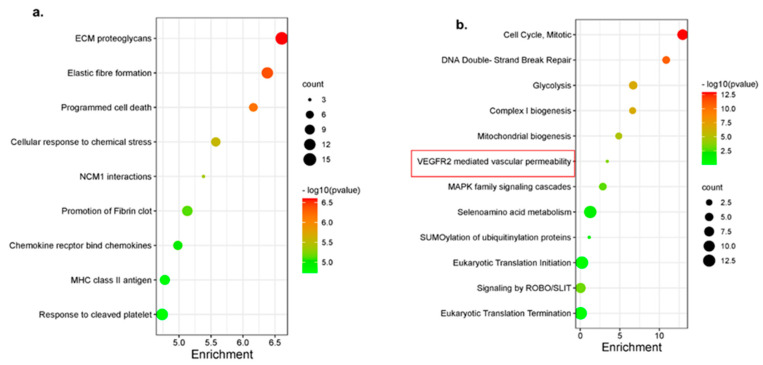
Pathway alterations upon Disarib treatment in EAC mouse tumour. (**a**) A bubble plot depicting upregulated pathways (**b**) A bubble plot depicting downregulated pathways. The *x*-axis represents enrichment and *y*-axis pathways. The size of the circle represents gene count, and the colouring is based on −log10 (*p*-value).

The significant upregulated and downregulated gene list (padj < 0.05, log2FC, 1, −1) was given as input to the Reactome database for EAC samples. ECM proteoglycans, MHC class II antigens, programmed cell death, chemokine signalling, and platelet response pathways were upregulated (Figure 3a). ABT-737 had thrombocytopenia as one of the side effects in patients. Unlike ABT-737, Disarib did not have an impact on platelets, platelet forming pathways are upregulated. Mitochondrial-associated pathways, glycolysis, cell cycle, VEGF-mediated angiogenesis, and MAPK signalling pathways that form the cancer hallmarks and promote tumour formation were downregulated upon Disarib treatment in EAC samples (Figure 3b). BCL2 is known to maintain mitochondrial membrane integrity and crosstalk with pathways like angiogenesis and MAPK pathway; therefore, when Disarib disrupts BCL2, mitochondrial processes and its other crosstalk pathway get affected and contribute towards tumour reduction.

### 3.5. EAC Tumour Mice Displayed High Levels of Oncometabolite Compared to Normal Animals

**Figure 4:**
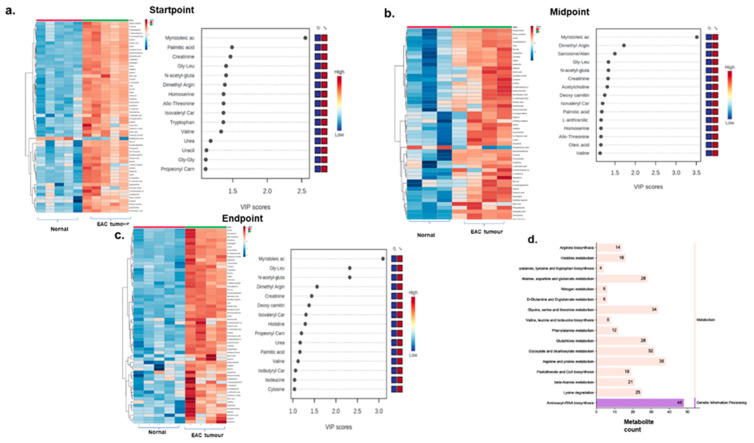
Metabolomics analysis of Normal and EAC tumour samples. Heatmaps depicting significant metabolites for all three-time points. The blue colour represents downregulation, and the brown colour represents upregulation. VIP score plot obtained from multivariate regression analysis. Top metabolites based on the VIP scores are plotted for (**a**) Startpoint (**b**) Midpoint and (**c**) Endpoint. The notation 0 and 1 represent normal and tumour samples, respectively. The blue colour represents low expression, and the red colour represents high expression. (**d**) Pathway enrichment analysis of significant metabolites. The *x*-axis represents metabolite count, and the *y*-axis represents pathways.

EAC tumour was injected in swiss albino mice. Once the tumour developed, Disarib was administered 50 mg/kg body weight, 12 doses, daily. Blood was collected from the tail vein at three different time points during the experiment: (a) before the treatment, (b) at the midpoint during the treatment, and (c) at the end of the experiment. Blood was collected from four different groups of animals: EAC tumour control, EAC tumour group receiving Disarib treatment, Normal animals, and Normal mice receiving Disarib treatment. Each group had 5 animals. Plasma was isolated from the blood of all the animals at three different time points and was injected into LC-MS. The peak intensities obtained after targeted metabolomic profiling of the mice samples were normalised based on the internal standards and log transformed. Normalised values of the metabolites were subjected to principal component analysis. An apparent clustering of normal vs. tumour control samples was observed (Supplementary Figure S2a). Differential analysis of normal animals and EAC tumour control animals was performed to understand the metabolomic profile of EAC tumours. A total of 65 metabolites were scored, of which 59 were significant for the start point, 52 were significant at the midpoint, and 59 were significant at the endpoint of the experiment (Supplementary Figure S2b), suggesting that there was no change in the levels of oncometabolite in all the three-time points in EAC tumour control animals (Figure 4a–c). Known oncometabolite such as carnitine and its derivatives, sarcosine, glycine, uracil, kynurenine, and oleic acid are known to drive cancer progression, and metastasis was observed in the EAC tumour control profile. Partial Least Squares—Discriminant Analysis (PLS-DA) utilises multivariate regression and extracts information through a linear combination of variables to predict classification. PLS-DA calculates the weighted sum of squares of the loadings and generates Variable Importance in Projection (VIP) scores for each metabolite. VIP scores ≥ 1 were plotted for each time point and analysed (Figure 4a–c). Results suggest that Myristoleic acid, an oncometabolite that participates in fatty acid metabolism and is known to promote tumorigenesis, had the highest VIP score at all time points in tumour animals. Tryptophan, known to drive cancer, has a high VIP score at the start but is absent as the tumour progresses. Oncometabolites sarcosine and homoserine are explicitly seen at the midpoint, and histidine with the highest VIP score at the endpoint. The other metabolites remain constant through all the time points. Pathway enrichment analysis revealed that oncometabolites from the tumour belonged to Aminoacyl-tRNA biosynthesis, Arginine biosynthesis, Phenylalanine, tyrosine, and tryptophan biosynthesis, purine metabolism, fatty acid metabolism, glutathione metabolism, histidine, and biotin metabolism (Figure 4d). Any change in the metabolites due to Disarib alone in the normal animals without tumours was subtracted.

### 3.6. Disarib Treatment Reduced Oncometabolite Levels in EAC Tumours

**Figure 5:**
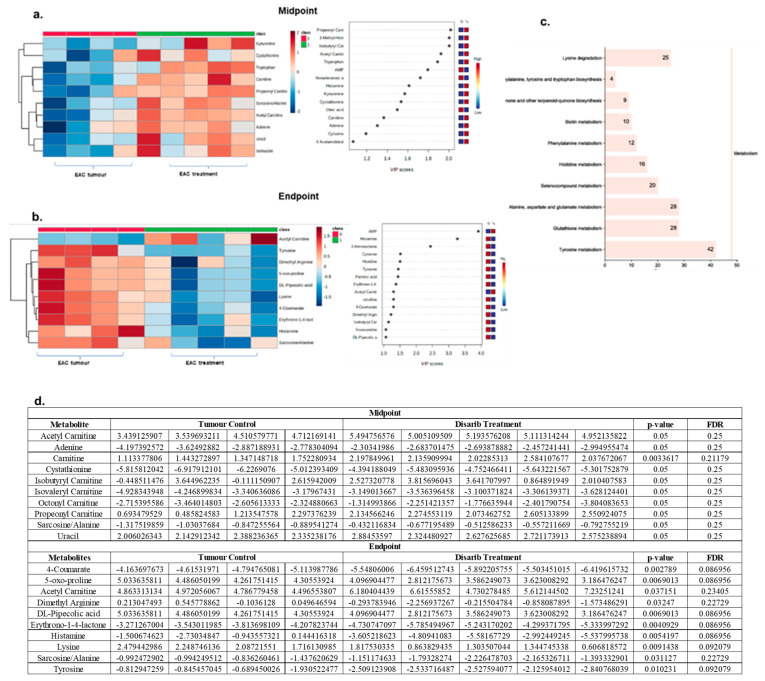
Metabolomics analysis of EAC tumour control and Disarib treated samples. Heatmaps depicting significant metabolites and VIP score plots were obtained from multivariate regression analysis. Top metabolites based on the VIP scores greater than are plotted for (**a**) Midpoint (**b**) Endpoint. Notations 0 and 1 represent tumour control and Disarib treated samples, respectively. The blue colour represents low expression, and the red colour represents high expression. (**c**) Pathway enrichment analysis of significant metabolites. The *x*-axis represents metabolite count, and the *y*-axis represents pathways. (**d**) Table showing normalized peak intensities, *p*-value and FDR for metabolites from midpoint and endpoint in tumour control and Disarib treated samples.

It was evident from the previous analysis that significant metabolites in tumour animals are representative of the oncogenic progression. It was followed up by Disarib treatment in tumour-bearing mice, with the aim of understanding the impact of Disarib on oncometabolites in EAC tumour mice. To begin with the metabolite analysis, the normalised peak intensities of metabolites were subjected to principal component analysis. Distinct segregation of tumour control and Disarib-treated samples was observed (Supplementary Figure S2c). Additionally, differential analysis of normal and normal treated with Disarib was performed to subtract the background metabolites from the tumour-treated samples. A total of 65 metabolites were scored in tumour vs. Disarib-treated samples. We performed univariate and multivariate analyses of the metabolites at the start, mid and endpoint of the study. Univariate analysis revealed 10 significant metabolites at the midpoint and 10 at the endpoint (Supplementary Figure S2d) of the experiment, suggesting a drastic reduction in the number of oncometabolite following Disarib treatment. Normalised peak intensities for these 10 significant metabolites are depicted in the heatmap for control and the Disarib treatment group for the mid-point (Figure 5a) and endpoint (Figure 5b). Additionally, the normalized peak intensity values along with *p*-value and FDR values are provided in Figure 5d. Although an initial increase in Oncometabolites like sarcosine was observed at the midpoint, Disarib-induced downregulation was evident at the endpoint, whereas levels of oncometabolites such as Dimethyl arginine, 5-oxo-proline and tyrosine were down upon Disarib treatment at the endpoint (Figure 5b). Also, when partial Least Squares—Discriminant Analysis (PLS-DA) was performed, VIP scores for each metabolite were generated via multivariate regression analysis. VIP scores ≥ 1 were plotted for each time point and analysed. At the start point of Disarib treatment, all the tumour oncometabolites were detected, as described previously. At the midpoint, the tumour is still pushing towards proliferation; therefore, oncometabolites such as carnitine, glycine, cytosine, and kynurenine are still high. However, a reduction in metabolites from the fatty acid metabolism was observed. It was interesting to observe that at the endpoint, tumour suppressor metabolites acetylcarnitine and Adenosine monophosphate increased, whereas the oncometabolites, cytosine, arginine, tyrosine, palmitic acid reduced, as revealed by multivariate analysis (Figure 5a,b). AMP, cytosine and histamine were the common metabolites obtained from both univariate and multivariate regression analysis. Pathway enrichment analysis of 10 significant metabolites at the endpoint with FDR ≤ 0.25 revealed that the downregulated oncometabolite upon Disarib treatment belonged to Aminoacyl-tRNA biosynthesis, lysine degradation, Phenylalanine, tyrosine, and tryptophan biosynthesis, Ubiquinone and another terpenoid-quinone biosynthesis, histidine and biotin metabolism pathways (Figure 5d). A statistical meta-analysis was performed between the timepoints using Metabonalyst software. The association analysis revealed Tryptophan, Acetyl Carnitine, Creatinine, N, N Dimethyl Glycine/Methyl alanine, and uracil as key metabolites associated with all the time points, and their levels changed significantly upon Disarib treatment, indicating the effect of Disarib on metabolism.

### 3.7. Integration of Transcriptome and Metabolome of Disarib Treated EAC Samples Revealed Altered Amino Acid and Purine Metabolism

**Figure 6:**
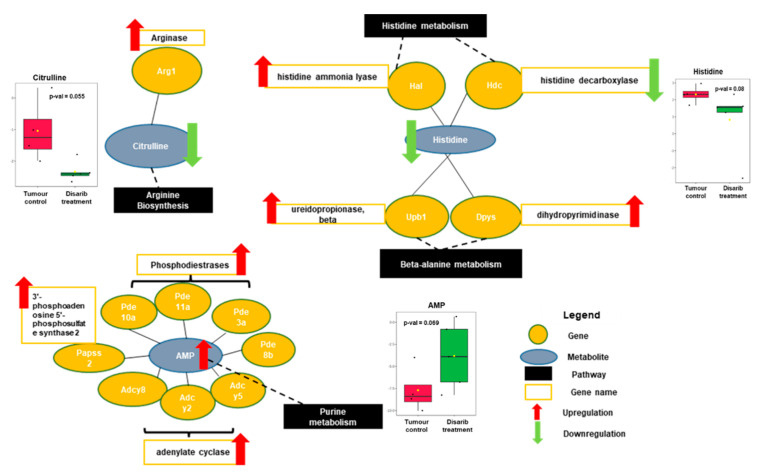
Interaction network between transcriptome and metabolomic data of Disarib treated EAC tumour samples. Significant DEGs and significant metabolites with VIP scores greater than 1 were utilized to generate the matched features using metaboanalyst. DEGs are represented in yellow circle, metabolites in grey and pathway in black rectangle. Red colour arrow denotes upregulation and green arrow denotes downregulation. Individual box plots with fold changes and *p*-value are plotted for citrulline, histidine and AMP metabolites where red colour denotes tumour control, and green colour denotes Disarib treated samples.

To understand if changes in the metabolites correlate with changes in the transcriptome, the significant gene list from Disarib-treated EAC tumour samples and the significant metabolite list with VIP scores greater than one at the endpoint was given input to metaboanalyst software. The joint pathway analysis option was chosen in metaboanalyst with the hypergeometric test as the enrichment factor and degree of centrality as the topological measure. The output was a list of pathways having both metabolite and corresponding genes. After data integration, amino acid metabolism (histidine and arginine), β-alanine metabolism, and purine metabolism were picked up (Figure 6). Histidine, an oncometabolite, is reduced following Disarib treatment and histidine amino lyase; the enzyme catalysing its degradation is upregulated, and histidine decarboxylase converts histidine to histamine is low. β-alanine is an intermediate of the pyrimidine degradation pathway. Upregulation of the Ureidopropionasebeta and dihydropyrimidinase enzymes involved in *β*-alanine metabolism was observed. *β*-alanine is known to control tumorigenesis by altering energy metabolism in cancer cells [56]. Citrulline and arginine promote tumour growth and angiogenesis, and their levels are downregulated after Disarib treatment, although the enzyme arginase—transcripts—are upregulated after Disarib treatment. The levels of cAMP decrease despite Adenylate Cyclase being upregulated due to the action of Phosphodiesterase (Figure 6). cAMP is known to regulate tumour cell proliferation by activating downstream targets [57]. Adenosine monophosphate (AMP) levels, a tumour suppressor metabolite, increased upon Disarib treatment at midpoint, driving the process of tumour regression most probably through AMPK activation. AMP regulates AMPK activity a known tumour suppressor [58]. Adenyl cyclase enzyme expression that converts ATP to cyclic AMP and the Phosphodiesterase enzyme that converts c-AMP back to AMP correlated with elevated levels of AMP in Disarib-treated tumour samples.

### 3.8. Disarib Significantly Reduces Angiogenesis in EAC Tumours

**Figure 7:**
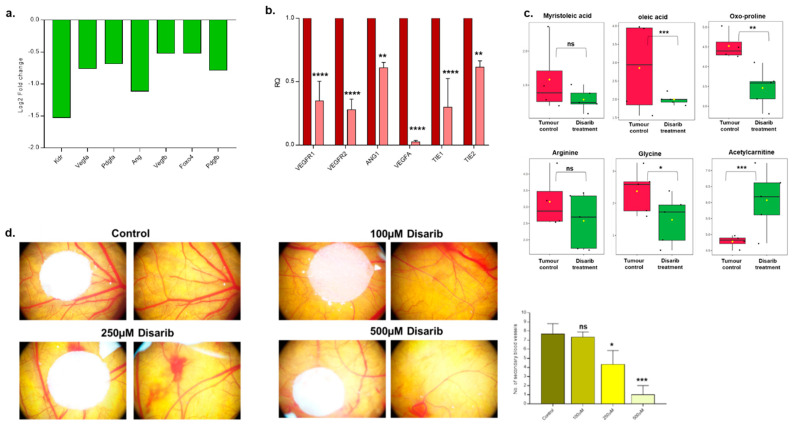
Anti-angiogenic effects of Disarib. (**a**) Line graph depicting expression of angiogenic markers in Disarib-treated EAC tumour model from RNAseq data. The *x*-axis represents genes, and the *y*-axis represents log2Foldchange. (**b**) Grouped bar graph showing expression of angiogenic markers in Disarib-treated EAC tumour model from qRTPCR. The maroon bar represents tumour control, and the pink bar represents Disarib-treated samples. The *x*-axis represents genes, and the *y*-axis represents relative quantification. (**c**) Individual box plots depict fold change of metabolites participating in angiogenesis. Red colour denotes tumour control, and green colour denotes Disarib-treated samples. (**d**) Anti-angiogenic effects of Disarib. Representative images of CAM assay. There are four panels: control, 100 µM, 250 µM and 500 µM Disarib (with and without the disc) and a bar graph showing the number of secondary blood vessels. The graph shows mean # SEM (ns: not significant, * *p* < 0.05, ** *p* < 0.005, *** *p* < 0.0001, **** *p* < 0.00001).

The pathway analysis of Disarib-treated EAC tumours showed that genes participating in angiogenesis were significantly downregulated. We wanted to investigate further whether Disarib possesses anti-angiogenic property. VEGF and its partners are significant players in angiogenesis and are upregulated in breast cancer [48]. Therefore, we checked for the expression of genes belonging to VEGF signalling in RNAseq data and plotted the graph. The results showed that the significant angiogenesis markers were downregulated with Disarib treatment (Figure 7a). A qRT-PCR was performed to validate the results from RNA-seq on the EAC tumour control and Disarib-treated samples. The qRT-PCR was performed for angiogenesis-related genes VEGFA, VEGFR1, VEGFR2, ANG1, TIE1, and TIE2. The Disarib-treated tumour samples showed decreased expression of angiogenic markers compared to control. Among the markers, the ligand VEGFA major player in angiogenesis and its receptors VEGFR1 and VEGFR2 showed maximum downregulation in the treated samples (Figure 7b). Metabolites participating in angiogenesis were also analysed. Analysis revealed metabolites from fatty acid metabolisms and amino acids that are pro-angiogenic and downregulated upon Disarib treatment (Figure 7c). Oleic acid, glycine and oxo-proline were significantly downregulated in Disarib treated samples compared to the tumour control. Acetyl carnitine, an anti-angiogenic metabolite, was significantly elevated in Disarib-treated plasma samples compared to the control (Figure 7c). Therefore, this suggests that at both transcriptomic and metabolomic levels, Disarib reduced angiogenic factors. Lastly, to further confirm the anti-angiogenic effect of Disarib, we performed a chick chorioallantoic membrane (CAM) assay. CAM is an extra-embryonic membrane comprised of a high density of blood and lymphatic vessels. CAM has a dense capillary network and is routinely used to study in vivo angiogenesis and anti-angiogenesis in response to potential biomolecules and drugs. A window was made on the three-day-old fertilised egg, and a disc with 100 µM, 250 µM, and 500 µM Disarib was placed on CAM on the fifth day, inside the egg, near the blood vessels. After 48 h, photographs were taken to observe any changes in the blood vessels. A minimum of five eggs was used for the control and treatment groups. Blood vessels were counted in all five eggs and plotted as a bar graph. A representative image for all the concentrations has been shown in Figure 7d. Results suggest a significant reduction in the number of secondary blood vessels in a concentration-dependent manner in Disarib-treated eggs compared to control. The highest concentration, 500 µM, showed the maximum reduction.

## 4. Discussion

This study explored the global gene expression and correlated it with changes in metabolites post-Disarib treatment in an EAC Breast cancer mouse model. A significant reduction of oncometabolite and upregulation of tumour suppressor metabolites led to tumour regression in vivo. Integrated transcriptome and metabolome led to the identification of the amino acid metabolism and angiogenesis, among other pathways to be altered upon Disarib treatment.

Breast cancer is a heterogeneous disease and involves the loss of tumour suppressors and amplification of oncogenes leading to activation of oncogenic signalling and tumorigenesis [1]. The Disarib-induced cell death may be due to a tilt in the balance of tumour suppressors and oncogenes in the EAC breast cancer mouse model. Disarib modulated 6 oncogenes and 101 TSGs. Global gene expression using RNAseq analysis has been utilised to predict drug resistance, chemotherapy, biomarker analysis, diagnosis, and drug development.

One of the major issues of previous Bcl2 inhibitors has been thrombocytopenia. ABT-737 and ABT-263 were withdrawn from the trials because they severely decreased platelet counts [59,60]. Interestingly, in Disarib-treated mice, platelet-related pathways were unperturbed. Programmed cell death, MHCII antigen, and chemokine signalling were upregulated in EAC tumours treated with Disarib. Oncogenic signalling pathways, DNA repair, cell cycle, metabolism, mitochondrial pathways and angiogenesis are upregulated in Breast cancer [61,62,63]. Disarib treatment reduced the levels of genes participating in the cancer hallmark pathways. Specifically, Disarib downregulated angiogenesis signalling. Angiogenesis is known to activate the EMT pathway and promote the migration of breast cancer. Therefore, we checked for the anti-angiogenic properties of Disarib. Disarib is derived from Z24 compounds that possess anti-angiogenic properties [25,64]. Bcl2 is involved in crosstalk with VEGF-mediated signalling, and overexpression of Bcl2 leads to increased angiogenesis [17,18,22,65]. TW37, a Bcl2 inhibitor, has shown anti-angiogenic properties [24]. Disarib blocked angiogenesis efficiently by breaking the interaction between Bcl2-Bak. VEGF and its receptors VEGFRs are major players in tumour angiogenesis [66]. VEGFA, VEGFR1, and VEGFR2 are upregulated in breast cancer [67,68,69,70]. High levels of Tie1 receptor are associated with poor survival in breast cancer [71]. All the angiogenesis markers were downregulated upon Disarib treatment, and a significant reduction in the number of secondary blood vessels was seen in the CAM assay confirming the anti-angiogenic effect of Disarib.

Cancer cells are known to modulate the energy metabolism pathways for their survival. The ultimate measure of the alterations in the phenotype can be attributed to the changes in the biochemical action in a cell due to a drug. The assay to detect the action of the metabolic pathways is to measure metabolites. We performed metabolomic profiling using targeted LC-MS on plasma samples of normal EAC tumour controls and Disarib-treated EAC tumour animals at different time points. For the positive mode, data were normalized using internal standard L-Zeatine, and L-Tryptophan was for the negative mode of acquisition. To take care of the batch effects, sample replicates from previous batches were injected and verified. Pooled plasma was employed as quality controls at the start, middle, and end of the batch run. EAC tumour control showed high levels of oncometabolite that remained constant throughout the tumour development. Kynurenine, Sarcosine, Myristoleic acid, Glycine, Dimethylarginine, and tryptophan are oncometabolite known to play a role in tumour progression [72,73,74,75,76,77]. Myristoleic acid promotes angiogenesis, and acetylcarnitine possesses anti-angiogenic properties [78,79,80]. These metabolites were seen in EAC tumour control samples.

ABT-199, an FDA-approved Bcl2 inhibitor, is known to cause resistance in leukemic cells by elevating levels of a metabolite—phosphoinositides—in cells exhibiting a high glycolytic rate [81]. Interestingly, we observed that Disarib treatment reduced oncometabolite like myristoleic acid, carnitine, and Citrulline and increased the levels of AMP and acetylcarnitine. Leucine and isoleucine degradation, glycine and serine metabolism, valine, glutathione metabolism, and purine metabolism have been observed in obese breast cancer patients when an integrated transcriptomics and metabolomics study was performed [16]. Histidine, arginine, and Citrulline are oncometabolite and known for cancer progression [82,83]. Histidine and Citrulline were downregulated upon Disarib treatment, and an integrated transcriptomic and metabolomic analysis revealed that enzymes belonging to these pathways were upregulated. The enzyme Arginase transcripts were upregulated whereas Arginine was downregulated. The downstream breakdown product of Arginine, NO vs. ornithine is essential to drive the macrophages in the tumour microenvironment. The role of NO in driving macrophages to a polarized state M1/M2 has a very important role to play in tumour progression/inhibition [84]. No significant change in the levels of ornithine was observed upon Disarib treatment, indicating a favourable condition which matched the phenotype tumour regression. CAMP is a known driver of tumours [85,86]. AMP is a tumour suppressor metabolite [87,88]. Integrated analysis revealed that AMP was upregulated and adenyl cyclase enzyme was upregulated upon Disarib treatment. Adenyl cyclase converts ATP to cyclic AMP, which provides energy to the tumour progression [89]. However, the phosphodiesterase enzyme is upregulated, converting cyclic AMP back to AMP [90]. The high levels of AMP correlated with elevated AC and PDE transcripts. Fatty acids like oleic acid, myristoleic acid, and amino acids like arginine and glycine are pro-angiogenic. Disarib treatment reduced the levels of pro-angiogenic metabolites. Acetyl carnitine, an anti-angiogenic metabolite, was elevated in Disarib-treated samples. The downregulation of the metabolites, which play a crucial role in energy metabolism and cell survival, cannot alone be deduced from the transcriptomic analysis. A combined metabolome and transcriptome provided a better understanding of changes induced by a Bcl2 inhibitor, Disarib.

## 5. Conclusions

Through transcriptomic analysis of Disarib-treated EAC mouse tumours, we obtained overall changes caused by Disarib. We identified potential pathways that cause resistance and thrombocytopenia to be downregulated. We confirmed that Bcl2 inhibition could potentially block angiogenesis. By integrating transcriptomics and metabolomics, we could infer that Disarib reduces tumours by activating apoptosis, regulating angiogenesis, and altering the levels of key oncometabolite.

## Data Availability

Not applicable.

## Supplementary Materials

The following supporting information can be downloaded at: https://www.mdpi.com/article/10.3390/genes13071208/s1, Table S1: Primers used for qRT-PCR validation; Table S2: Sequencing details of EAC samples; Figure S1: Oncogene and tumour suppressor gene analysis; Figure S2: Metabolomics analysis of Normal and EAC tumour samples; File S1: QQQ Check Tune Report; File S2: Quality control and Multiple reaction monitoring results of detected metabolites; File S3: Differential gene expression table for EAC tumour control vs. EAC tumour treated with Disarib.

## References

[1] Sawyers C. (2004). Targeted cancer therapy. Nature.

[2] Bouchalova K., Svoboda M., Kharaishvili G., Radova L., Bouchal J., Trojanec R., Koudelakova V., Hajduch M., Cwiertka K., Kolar Z. (2012). BCL2 Protein in Prediction of Relapse in Triple-Negative Breast Cancer (TNBC) Treated with Adjuvant Anthracycline-Based Chemotherapy.

[3] Bhargava V., Kell D.L., van de Rijn M., Warnke R.A. (1994). Bcl-2 immunoreactivity in breast carcinoma correlates with hormone receptor positivity. Am. J. Pathol..

[4] Delbridge A.R., Grabow S., Strasser A., Vaux D.L. (2016). Thirty years of BCL-2: Translating cell death discoveries into novel cancer therapies. Nat. Rev. Cancer.

[5] Radha G., Raghavan S.C. (2017). BCL2: A promising cancer therapeutic target. Biochim. Biophys. Acta (BBA) Rev. Cancer.

[6] Souers A.J., Leverson J.D., Boghaert E.R., Ackler S.L., Catron N.D., Chen J., Dayton B.D., Ding H., Enschede S.H., Fairbrother W.J. (2013). ABT-199, a potent and selective BCL-2 inhibitor, achieves antitumor activity while sparing platelets. Nat. Med..

[7] Tahir S.K., Smith M.L., Hessler P., Rapp L.R., Idler K.B., Park C.H., Leverson J.D., Lam L.T. (2017). Potential mechanisms of resistance to venetoclax and strategies to circumvent it. BMC Cancer.

[8] Choudhary G.S., Al-Harbi S., Mazumder S., Hill B.T., Smith M.R., Bodo J., Hsi E.D., Almasan A. (2015). MCL-1 and BCL-xL-dependent resistance to the BCL-2 inhibitor ABT-199 can be overcome by preventing PI3K/AKT/mTOR activation in lymphoid malignancies. Cell Death Dis..

[9] Bose P., Gandhi V., Konopleva M. (2017). Pathways and mechanisms of venetoclax resistance. Leuk. Lymphoma.

[10] Vartak S.V., Iyer D., Santhoshkumar T.R., Sharma S., Mishra A., Goldsmith G., Srivastava M., Srivastava S., Karki S.S., Surolia A. (2017). Novel BCL2 inhibitor, Disarib induces apoptosis by disruption of BCL2-BAK interaction. Biochem. Pharmacol..

[11] Sharma S., Varsha K.K., Kumari S., Gopalakrishnan V., Jose A.E., Choudhary B., Mantelingu K., Raghavan S.C. (2020). Acute toxicity analysis of Disarib, an inhibitor of BCL2. Sci. Rep..

[12] Haas B.J., Zody M.C. (2010). Advancing RNA-seq analysis. Nat. Biotechnol..

[13] Subramanian A., Narayan R., Corsello S.M., Peck D.D., Natoli T.E., Lu X., Gould J., Davis J.F., Tubelli A.A., Asiedu J.K. (2017). A next generation connectivity map: L1000 platform and the first 1,000,000 profiles. Cell.

[14] Beger R.D. (2013). A review of applications of metabolomics in cancer. Metabolites.

[15] Vermeersch K.A., Styczynski M.P. (2013). Applications of metabolomics in cancer research. J. Carcinog..

[16] Hassan M.A., Al-Sakkaf K., Shait Mohammed M.R., Dallol A., Al-Maghrabi J., Aldahlawi A., Ashoor S., Maamra M., Ragoussis J., Wu W. (2020). Integration of transcriptome and metabolome provides unique insights to pathways associated with obese breast cancer patients. Front. Oncol..

[17] Iervolino A., Trisciuoglio D., Ribatti D., Candiloro A., Biroccio A., Zupi G., Del Bufalo D. (2002). Bcl-2 overexpression in human melanoma cells increases angiogenesis through VEGF mRNA stabilization and HIF-1mediated transcriptional activity. FASEB J..

[18] Karl E., Zhang Z., Dong Z., Neiva K.G., Soengas M.S., Koch A.E., Polverini P.J., Nunez G., Nör J.E. (2007). Unidirectional crosstalk between Bcl-xL and Bcl-2 enhances the angiogenic phenotype of endothelial cells. Cell Death Differ..

[19] Tonini T., Rossi F., Claudio P.P. (2003). Molecular basis of angiogenesis and cancer. Oncogene.

[20] Carmeliet P., Jain R.K. (2000). Angiogenesis in cancer and other diseases. Nature.

[21] Fantozzi A., Gruber D.C., Pisarsky L., Heck C., Kunita A., Yilmaz M., Meyer-Schaller N., Cornille K., Hopfer U., Bentires-Alj M. (2014). VEGF-mediated angiogenesis links EMT-induced cancer stemness to tumor initiation. Cancer Res..

[22] Hicklin D.J., Ellis L.M. (2005). Role of the vascular endothelial growth factor pathway in tumor growth and angiogenesis. J. Clin. Oncol..

[23] Deshayes F., Nahmias C. (2005). Angiotensin receptors: A new role in cancer?. Trends Endocrinol. Metab..

[24] Zeitlin B.D., Joo E., Dong Z., Warner K., Wang G., Nikolovska-Coleska Z., Wang S., Nör J.E. (2006). Antiangiogenic effect of TW37, a small-molecule inhibitor of Bcl-2. Cancer Res..

[25] Iyer D., Vartak S.V., Mishra A., Goldsmith G., Kumar S., Srivastava M., Hegde M., Gopalakrishnan V., Glenn M., Velusamy M. (2016). Identification of a novel BCL2-specific inhibitor that binds predominantly to the BH1 domain. FEBS J..

[26] Geran R.I., Greenberg N.H., Macdonald M.M., Schumacher A.M., Abbott B.J. (1972). Protocols for screening chemical agents and natural products against animal tumors and other biological systems. Cancer Chemother. Rep..

[27] Noaman E., El-Din N.K.B., Bibars M.A., Abou Mossallam A.A., Ghoneum M. (2008). Antioxidant potential by arabinoxylan rice bran, MGN-3/biobran, represents a mechanism for its oncostatic effect against murine solid Ehrlich carcinoma. Cancer Lett..

[28] Wingett S.W., Andrews S. (2018). FastQ Screen: A tool for multi-genome mapping and quality control. F1000Research.

[29] Langmead B., Salzberg S.L. (2012). Fast gapped-read alignment with Bowtie 2. Nat. Methods.

[30] Li H., Handsaker B., Wysoker A., Fennell T., Ruan J., Homer N., Marth G., Abecasis G., Durbin R. (2009). The sequence alignment/map format and SAMtools. Bioinformatics.

[31] Quinlan A.R., Hall I.M. (2010). BEDTools: A flexible suite of utilities for comparing genomic features. Bioinformatics.

[32] Morvan M.L., Vert J.-P. (2017). Supervised quantile normalisation. arXiv.

[33] Peng R.D. (2016). R Programming for Data Science.

[34] Mortazavi A., Williams B.A., McCue K., Schaeffer L., Wold B. (2008). Mapping and quantifying mammalian transcriptomes by RNA-Seq. Nat. Methods.

[35] Croft D., O’kelly G., Wu G., Haw R., Gillespie M., Matthews L., Caudy M., Garapati P., Gopinath G., Jassal B. (2010). Reactome: A database of reactions, pathways and biological processes. Nucleic Acids Res..

[36] Wickham H. (2011). ggplot2. Wiley Interdiscip. Rev. Comput. Stat..

[37] Manual I. (2017). ProtoScript® First Strand cDNA Synthesis Kit. https://www.neb.com/-/media/nebus/files/manuals/manuale6550.pdf?rev=9c51e082b8614a27b3854e503273db87&hash=8BF8FA6D0CE9F0821F865C5250D22CB5.

[38] Mohr S., Ghanem E., Smith W., Sheeter D., Qin Y., King O., Polioudakis D., Iyer V.R., Hunicke-Smith S., Swamy S. (2013). Thermostable group II intron reverse transcriptase fusion proteins and their use in cDNA synthesis and next-generation RNA sequencing. Rna.

[39] Tzanetakis I.E., Keller K.E., Martin R.R. (2005). The use of reverse transcriptase for efficient first-and second-strand cDNA synthesis from single-and double-stranded RNA templates. J. Virol. Methods.

[40] Ponchel F., Toomes C., Bransfield K., Leong F.T., Douglas S.H., Field S.L., Bell S.M., Combaret V., Puisieux A., Mighell A.J. (2003). Real-time PCR based on SYBR-Green I fluorescence: An alternative to the TaqMan assay for a relative quantification of gene rearrangements, gene amplifications and micro gene deletions. BMC Biotechnol..

[41] Deepak S.A., Kottapalli K.R., Rakwal R., Oros G., Rangappa K.S., Iwahashi H., Masuo Y., Agrawal G.K. (2007). Real-time PCR: Revolutionizing detection and expression analysis of genes. Curr. Genom..

[42] Schmittgen T.D. (2001). Real-time quantitative PCR. Methods.

[43] Livak K.J., Schmittgen T.D. (2001). Analysis of relative gene expression data using real-time quantitative PCR and the 2- ΔΔCT method. Methods.

[44] Schmittgen T.D., Livak K.J. (2008). Analyzing real-time PCR data by the comparative CT method. Nat. Protoc..

[45] Swift M.L. (1997). GraphPad prism, data analysis, and scientific graphing. J. Chem. Inf. Comput. Sci..

[46] Lindahl A., Sääf S., Lehtiö J., Nordström A. (2017). Tuning metabolome coverage in reversed phase LC–MS metabolomics of MeOH extracted samples using the reconstitution solvent composition. Anal. Chem..

[47] Want E.J., O’Maille G., Smith C.A., Brandon T.R., Uritboonthai W., Qin C., Trauger S.A., Siuzdak G. (2006). Solvent-dependent metabolite distribution, clustering, and protein extraction for serum profiling with mass spectrometry. Anal. Chem..

[48] Pulukool S.K., Bhagavatham S.K.S., Kannan V., Sukumar P., Dandamudi R.B., Ghaisas S., Kunchala H., Saieesh D., Naik A.A., Pargaonkar A. (2021). Elevated dimethylarginine, ATP, cytokines, metabolic remodeling involving tryptophan metabolism and potential microglial inflammation characterize primary open angle glaucoma. Sci. Rep..

[49] Bhagavatham S.K.S., Khanchandani P., Kannan V., Potikuri D., Sridharan D., Pulukool S.K., Naik A.A., Dandamudi R.B., Divi S.M., Pargaonkar A. (2021). Adenosine deaminase modulates metabolic remodeling and orchestrates joint destruction in rheumatoid arthritis. Sci. Rep..

[50] Borg D., Tverdovsky A., Stripp R. (2017). A fast and comprehensive analysis of 32 synthetic cannabinoids using agilent triple quadrupole LC–MS-MS. J. Anal. Toxicol..

[51] Xiao J.F., Zhou B., Ressom H.W. (2012). Metabolite identification and quantitation in LC-MS/MS-based metabolomics. TrAC Trends Anal. Chem..

[52] Xia J., Wishart D.S. (2011). Web-based inference of biological patterns, functions and pathways from metabolomic data using MetaboAnalyst. Nat. Protoc..

[53] Kalivodová A., Hron K., Filzmoser P., Najdekr L., Janečková H., Adam T. (2015). PLS-DA for compositional data with application to metabolomics. J. Chemom..

[54] Ribatti D. (2017). The chick embryo chorioallantoic membrane (CAM) assay. Reprod. Toxicol..

[55] Tufan A.C., Satiroglu-Tufan N.L. (2005). The chick embryo chorioallantoic membrane as a model system for the study of tumor angiogenesis, invasion and development of anti-angiogenic agents. Curr. Cancer Drug Targets.

[56] Vaughan R.A., Gannon N.P., Garcia-Smith R., Licon-Munoz Y., Barberena M.A., Bisoffi M., Trujillo K.A. (2014). β-alanine suppresses malignant breast epithelial cell aggressiveness through alterations in metabolism and cellular acidity in vitro. Mol. Cancer.

[57] Stork P.J., Schmitt J.M. (2002). Crosstalk between cAMP and MAP kinase signaling in the regulation of cell proliferation. Trends Cell Biol..

[58] Kim J., Yang G., Kim Y., Kim J., Ha J. (2016). AMPK activators: Mechanisms of action and physiological activities. Exp. Mol. Med..

[59] Vandenberg C.J., Cory S. (2013). ABT-199, a new Bcl-2–specific BH3 mimetic, has in vivo efficacy against aggressive Myc-driven mouse lymphomas without provoking thrombocytopenia. Blood J. Am. Soc. Hematol..

[60] Cang S., Iragavarapu C., Savooji J., Song Y., Liu D. (2015). ABT-199 (venetoclax) and BCL-2 inhibitors in clinical development. J. Hematol. Oncol..

[61] Sledge G.W., Miller K.D. (2003). Exploiting the hallmarks of cancer: The future conquest of breast cancer. Eur. J. Cancer.

[62] Majidinia M., Yousefi B. (2017). DNA repair and damage pathways in breast cancer development and therapy. DNA Repair.

[63] Caldon C.E., Daly R.J., Sutherland R.L., Musgrove E.A. (2006). Cell cycle control in breast cancer cells. J. Cell. Biochem..

[64] Wang L., Li J., Zheng Z., Liu H., Du G., Li S. (2004). Antitumor activities of a novel indolin-2-ketone compound, Z24: More potent inhibition on bFGF-induced angiogenesis and bcl-2 over-expressing cancer cells. Eur. J. Pharmacol..

[65] Zeitlin B.D., Nör J.E. (2010). Small-molecule inhibitors reveal a new function for Bcl-2 as a proangiogenic signaling molecule. Small Mol. Inhib. Protein Protein Interact..

[66] Harmey J.H., Bouchier-Hayes D. (2002). Vascular endothelial growth factor (VEGF), a survival factor for tumour cells: Implications for anti-angiogenic therapy. Bioessays.

[67] Khosravi Shahi P., Soria Lovelle A., Perez Manga G. (2009). Tumoral angiogenesis and breast cancer. Clin. Transl. Oncol..

[68] Longatto Filho A., Lopes J.M., Schmitt F.C. (2010). Angiogenesis and breast cancer. J. Oncol..

[69] Zhu X., Zhou W. (2015). The emerging regulation of VEGFR-2 in triple-negative breast cancer. Front. Endocrinol..

[70] Yan J.-D., Liu Y., Zhang Z.-Y., Liu G.-Y., Xu J.-H., Liu L.-Y., Hu Y.-M. (2015). Expression and prognostic significance of VEGFR-2 in breast cancer. Pathol. Res. Pract..

[71] Tiainen L., Korhonen E.A., Leppänen V.-M., Luukkaala T., Hämäläinen M., Tanner M., Lahdenperä O., Vihinen P., Jukkola A., Karihtala P. (2019). High baseline Tie1 level predicts poor survival in metastatic breast cancer. BMC Cancer.

[72] Sreekumar A., Poisson L.M., Rajendiran T.M., Khan A.P., Cao Q., Yu J., Laxman B., Mehra R., Lonigro R.J., Li Y. (2009). Metabolomic profiles delineate potential role for sarcosine in prostate cancer progression. Nature.

[73] Chen J.-Y., Li C.-F., Kuo C.-C., Tsai K.K., Hou M.-F., Hung W.-C. (2014). Cancer/stroma interplay via cyclooxygenase-2 and indoleamine 2,3-dioxygenase promotes breast cancer progression. Breast Cancer Res..

[74] Morettin A., Baldwin R.M., Côté J. (2015). Arginine methyltransferases as novel therapeutic targets for breast cancer. Mutagenesis.

[75] Carracedo A., Cantley L.C., Pandolfi P.P. (2013). Cancer metabolism: Fatty acid oxidation in the limelight. Nat. Rev. Cancer.

[76] Menendez J.A., Lupu R. (2007). Fatty acid synthase and the lipogenic phenotype in cancer pathogenesis. Nat. Rev. Cancer.

[77] Khatami F., Aghamir S.M.K., Tavangar S.M. (2019). Oncometabolites: A new insight for oncology. Mol. Genet. Genom. Med..

[78] Samson F.P., Patrick A.T., Fabunmi T.E., Yahaya M.F., Madu J., He W., Sripathi S.R., Tyndall J., Raji H., Jee D. (2020). Oleic acid, cholesterol, and linoleic acid as angiogenesis initiators. ACS omega.

[79] Albini A., Bruno A., Baci D., Gallazzi M., Tramacere M. (2019). Acetyl-L-carnitine (ALCAR) inhibits angiogenesis, migration and macrophage recruitment in prostatic cancer cells. Cancer Res..

[80] Baci D., Bruno A., Bassani B., Tramacere M., Mortara L., Albini A., Noonan D.M. (2018). Acetyl-l-carnitine is an anti-angiogenic agent targeting the VEGFR2 and CXCR4 pathways. Cancer Lett..

[81] Alkhatabi H.A., Zohny S.F., Shait Mohammed M.R., Choudhry H., Rehan M., Ahmad A., Ahmed F., Khan M.I. (2022). Venetoclax-Resistant MV4-11 Leukemic Cells Activate PI3K/AKT Pathway for Metabolic Reprogramming and Redox Adaptation for Survival. Antioxidants.

[82] Sun C., Li T., Song X., Huang L., Zang Q., Xu J., Bi N., Jiao G., Hao Y., Chen Y. (2019). Spatially resolved metabolomics to discover tumor-associated metabolic alterations. Proc. Natl. Acad. Sci. USA.

[83] Feun L., You M., Wu C.J., Kuo M.T., Wangpaichitr M., Spector S., Savaraj N. (2008). Arginine deprivation as a targeted therapy for cancer. Curr. Pharm. Des..

[84] Rath M., Müller I., Kropf P., Closs E.I., Munder M. (2014). Metabolism via arginase or nitric oxide synthase: Two competing arginine pathways in macrophages. Front. Immunol..

[85] Fagin J.A., Petrini J.H. (2020). Oncogene-induced DNA damage: Cyclic AMP steps into the ring. J. Clin. Investig..

[86] Zhang H., Kong Q., Wang J., Jiang Y., Hua H. (2020). Complex roles of cAMP–PKA–CREB signaling in cancer. Exp. Hematol. Oncol..

[87] Faubert B., Vincent E.E., Poffenberger M.C., Jones R.G. (2015). The AMP-activated protein kinase (AMPK) and cancer: Many faces of a metabolic regulator. Cancer Lett..

[88] Shaw J.H., Wolfe R.R. (1987). Fatty acid and glycerol kinetics in septic patients and in patients with gastrointestinal cancer. The response to glucose infusion and parenteral feeding. Ann. Surg..

[89] Yu X.-F., Ni Q.-C., Chen J.-P., Xu J.-F., Jiang Y., Yang S.-Y., Ma J., Gu X.-L., Wang H., Wang Y.-Y. (2014). Knocking down the expression of adenylate cyclase-associated protein 1 inhibits the proliferation and migration of breast cancer cells. Exp. Mol. Pathol..

[90] Stroop S.D., Beavo J.A. (1991). Structure and function studies of the cGMP-stimulated phosphodiesterase. J. Biol. Chem..

