# Supplementary material for: Integrated Transcriptome and Metabolomic Analysis Reveal Anti-Angiogenic Properties of Disarib, a Novel Bcl2-Specific Inhibitor
Source: Genes (Basel). 2022 Jul 6;13(7):1208. doi: 10.3390/genes13071208 (PMC9316176; doi:10.3390/genes13071208)
Supplement: Supplementary file 1 [file genes-13-01208-s001.zip › Supplementary Document.pdf]

Supplementary Tables

Table S1: Primers used for qRT-PCR validation

Gene	Forward (5'-3')	Reverse (5'-3')	Species
GAPDH	CCCTTCATTGACCTCAACTACAT	CTGGAGATGGTGATGGGATTT	Human and mouse
VEGFA	CTCCAGGGCTTCATCGTTA	CAGAAGGAGAGCAGAAGTCC	Mouse
VEGFB	TGCCCATGAGTTCCATGC	CCCAGTTTGATGGCCCCA	Mouse
VEGFR1	GCTGCTTGGAGATCTCACTG	CAGCAGCTCAAGTGTCACC	Mouse
VEGFR2	TTCCAGATGCTGGGCAAGTC	ATGACATCTTGATTGTGGCAT	Mouse
TIE1	AATGGCAGACCAGGCAATC	CCCCACTGGTCTCCTTTAG	Mouse
TIE2	GTTGACTCTAGCTCGGACTGT	GAAGTCGAGAGGCGAT CCC	Mouse
ANG1	GCAAAGGCTGATAAGGTTATGA	AGCTACCAACAACAACAGCA	Mouse
ANG2	TTCTTCTTTACGGATAGCAAC	AGCCACGGTCAACAACTCGC	Mouse

Table S2: Sequencing details of EAC samples

Sample	Total reads	Mapped reads	Percent alignment
Control 1	53206560	44512637	83.66
Control 2	71524122	56752290	79.35
Disarib 1	66572053	56173044	84.38
Disarib 2	66434413	50095862	75.41

Supplementary Figures

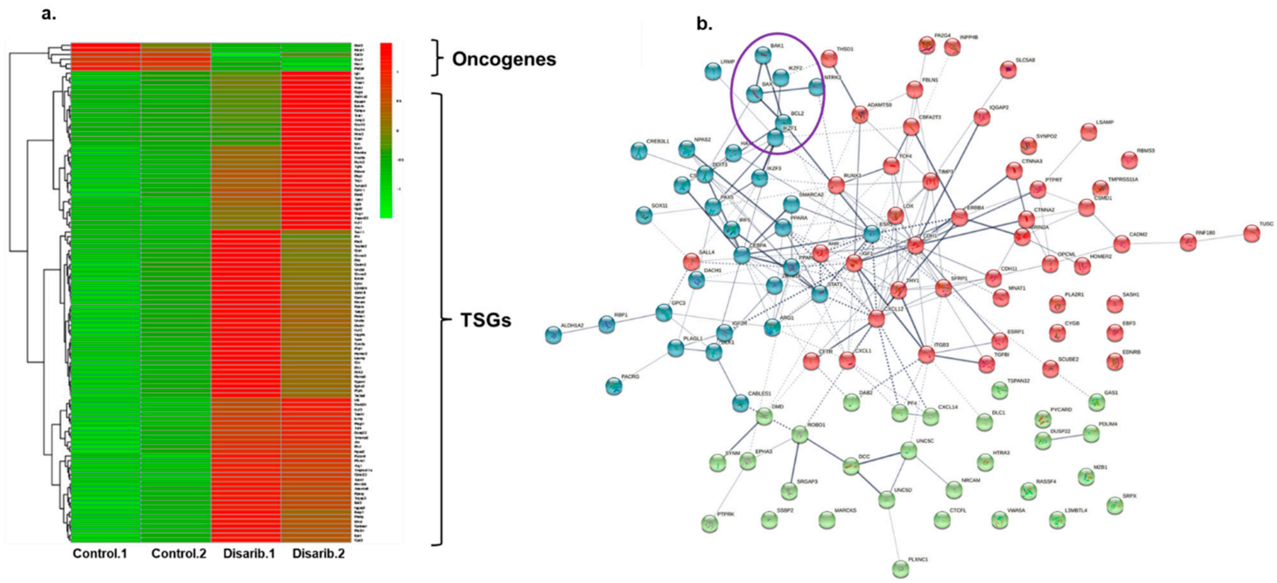

Figure S1: Oncogene and tumour suppressor gene analysis. **a.**Heatmap of oncogenes and tumour suppressor genes (TSGs) modulated Disarib in EAC tumour samples. Red colour indicates upregulation and green colour indicates downregulation. Heatmap is plotted based on row scaling and RPKM values. **b.** STRING interaction network of oncogenes and tumour suppressor genes interacting with BCL2, BAK in Disarib treated EAC tumour samples**.** The thickness of the line indicates the confidence of the interaction. The network has three colours pointing out 3 clusters obtained after k means clustering.

a.

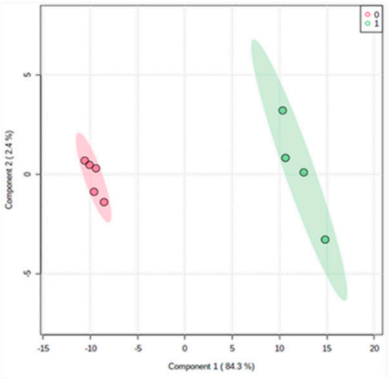

b.

Normal Vs EAC Tumour	Total	Significant
Startpoint	65	59
Midpoint	65	52
Endpoint	65	59

c.

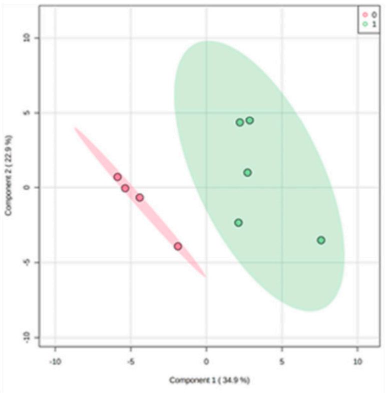

d.

EAC Tumour vs Disarib treatment	Total	Significcant
Midpoint	65	10
Endpoint	65	10

Figure S2: Metabolomics analysis of Normal and EAC tumour samples. **a**. A table depicting the number of total and significant metabolites **b.** Principal component analysis of Normal and EAC tumour samples. Red circles represent normal samples and the green circle represents tumour samples. Metabolomics analysis of EAC tumour and Disarib treated samples. **c.** A table depicting the number of total and significant metabolites **d.** Principal component analysis of EAC tumour and Disarib treated samples. Red circles represent tumour controls and the green circle represents Disarib treated samples.
