# Supplementary material for: Integrated Transcriptome and Metabolomic Analysis Reveal Anti-Angiogenic Properties of Disarib, a Novel Bcl2-Specific Inhibitor
Source: Genes (Basel). 2022 Jul 6;13(7):1208. doi: 10.3390/genes13071208 (PMC9316176; doi:10.3390/genes13071208)
Supplement: Supplementary file 1 [file genes-13-01208-s001.zip › Supplementary file S1.pdf]

QQQ Check Tune Report

**Instrument Name**	LC TQ
**MS Model**	G6490A
**MS Instrument Serial**	SG1402A201
**Software_Firmware Version**	10.0.127, FW: A.00.08.100
**Tune Date & Time**	11 October 2021 16:04:12
**Data Path**	D:\MassHunter\Tune\QQQ\G6490A\atunes.tune.xml
**Ion Source**	AJS ESI
**Ionization Mode**	AJS ESI
**Tuned Resolution**	All
**Vacuum Pressure**	2.71E+0 [R] (Torr); 5.28E-5 [H] (Torr)

Source Parameters

Parameter	Value
Gas Temp (°C)	220
Gas Flow (l/min)	19
Nebulizer (psi)	20
Capillary (V)	3000
Nozzle Voltage (V)	1500
Sheath Gas Temp (°C)	250
Sheath Gas Flow (l/min)	11

Positive Results

Analyzer: MS1 Polarity: Positive Width: Enhanced

m/z Expected	m/z Measured	Delta	Result	FWHM Expected	FWHM Measured	Delta	Result	Abundance
118.09	118.10	0.01	Pass	0.40	0.38	-0.02	Pass	857279
322.05	322.02	-0.03	Pass	0.40	0.39	-0.01	Pass	754000
622.03	621.97	-0.06	Pass	0.40	0.38	-0.02	Pass	183764
922.01	921.96	-0.05	Pass	0.40	0.41	0.01	Pass	78574
1221.99	1221.93	-0.06	Pass	0.40	0.38	-0.02	Pass	29515

Analyzer: MS2 Polarity: Positive Width: Enhanced

m/z Expected	m/z Measured	Delta	Result	FWHM Expected	FWHM Measured	Delta	Result	Abundance
118.09	118.05	-0.04	Pass	0.40	0.41	0.01	Pass	650383
322.05	321.99	-0.06	Pass	0.40	0.48	0.08	Pass	716870
622.03	621.90	-0.13	Pass	0.40	0.43	0.03	Pass	150450
922.01	921.88	-0.13	Pass	0.40	0.39	-0.01	Pass	55710
1221.99	1221.85	-0.14	Pass	0.40	0.37	-0.03	Pass	4202

Analyzer: MS1 Polarity: Positive Width: Unit

m/z Expected	m/z Measured	Delta	Result	FWHM Expected	FWHM Measured	Delta	Result	Abundance
118.09	118.09	0.00	Pass	0.70	0.71	0.01	Pass	1369258
322.05	322.04	-0.01	Pass	0.70	0.65	-0.05	Pass	1930298
622.03	622.01	-0.02	Pass	0.70	0.72	0.02	Pass	467414
922.01	921.96	-0.05	Pass	0.70	0.58	-0.12	Pass	284125
1221.99	1221.90	-0.09	Pass	0.70	0.69	-0.01	Pass	117151

Analyzer: MS2 Polarity: Positive Width: Unit

m/z Expected	m/z Measured	Delta	Result	FWHM Expected	FWHM Measured	Delta	Result	Abundance
118.09	118.09	0.00	Pass	0.70	0.69	-0.01	Pass	1714792
322.05	322.00	-0.05	Pass	0.70	0.70	0.00	Pass	1574553
622.03	622.01	-0.02	Pass	0.70	0.56	-0.14	Pass	480735
922.01	921.99	-0.02	Pass	0.70	0.71	0.01	Pass	335299
1221.99	1221.87	-0.12	Pass	0.70	0.57	-0.13	Pass	119559

Analyzer: MS1 Polarity: Positive Width: Wide

m/z Expected	m/z Measured	Delta	Result	FWHM Expected	FWHM Measured	Delta	Result	Abundance
118.09	118.10	0.01	Pass	1.20	1.17	-0.03	Pass	2007739
322.05	322.07	0.02	Pass	1.20	1.20	0.00	Pass	2501943
622.03	622.08	0.05	Pass	1.20	1.08	-0.12	Pass	710805
922.01	921.97	-0.04	Pass	1.20	1.19	-0.01	Pass	374008
1221.99	1221.99	0.00	Pass	1.20	1.11	-0.09	Pass	214295

Analyzer: MS2 Polarity: Positive Width: Wide

m/z Expected	m/z Measured	Delta	Result	FWHM Expected	FWHM Measured	Delta	Result	Abundance
118.09	118.07	-0.02	Pass	1.20	1.22	0.02	Pass	2290622
322.05	321.99	-0.06	Pass	1.20	1.29	0.09	Pass	2529635
622.03	622.06	0.03	Pass	1.20	1.16	-0.04	Pass	1001589
922.01	921.97	-0.04	Pass	1.20	1.18	-0.02	Pass	873203
1221.99	1221.88	-0.11	Pass	1.20	1.37	0.17	Pass	439751

QQQ Check Tune Report

Negative Results

Analyzer: MS1 Polarity: Negative Width: Enhanced

m/z Expected	m/z Measured	Delta	Result	FWHM Expected	FWHM Measured	Delta	Result	Abundance
112.99	112.98	-0.01	Pass	0.40	0.39	-0.01	Pass	206169
302.00	301.97	-0.03	Pass	0.40	0.38	-0.02	Pass	124720
601.98	601.94	-0.04	Pass	0.40	0.40	0.00	Pass	259186
1033.99	1033.94	-0.05	Pass	0.40	0.40	0.00	Pass	271435
1333.97	1333.88	-0.09	Pass	0.40	0.40	0.00	Pass	237297

Analyzer: MS2 Polarity: Negative Width: Enhanced

m/z Expected	m/z Measured	Delta	Result	FWHM Expected	FWHM Measured	Delta	Result	Abundance
112.99	112.98	-0.01	Pass	0.40	0.42	0.02	Pass	225470
302.00	301.97	-0.03	Pass	0.40	0.42	0.02	Pass	122855
601.98	602.04	0.06	Pass	0.40	0.38	-0.02	Pass	213057
1033.99	1034.09	0.10	Pass	0.40	0.36	-0.04	Pass	94753
1333.97	1334.06	0.09	Pass	0.40	0.37	-0.03	Pass	49932

Analyzer: MS1 Polarity: Negative Width: Unit

m/z Expected	m/z Measured	Delta	Result	FWHM Expected	FWHM Measured	Delta	Result	Abundance
112.99	112.95	-0.04	Pass	0.70	0.65	-0.05	Pass	365457
302.00	302.01	0.01	Pass	0.70	0.66	-0.04	Pass	259940
601.98	601.90	-0.08	Pass	0.70	0.68	-0.02	Pass	563954
1033.99	1033.91	-0.08	Pass	0.70	0.66	-0.04	Pass	1161972
1333.97	1333.81	-0.16	Pass	0.70	0.63	-0.07	Pass	896013

Analyzer: MS2 Polarity: Negative Width: Unit

m/z Expected	m/z Measured	Delta	Result	FWHM Expected	FWHM Measured	Delta	Result	Abundance
112.99	112.96	-0.03	Pass	0.70	0.71	0.01	Pass	486042
302.00	302.01	0.01	Pass	0.70	0.65	-0.05	Pass	337215
601.98	602.01	0.03	Pass	0.70	0.57	-0.13	Pass	659732
1033.99	1034.11	0.12	Pass	0.70	0.54	-0.16	Pass	764197
1333.97	1334.10	0.13	Pass	0.70	0.57	-0.13	Pass	880805

Analyzer: MS1 Polarity: Negative Width: Wide

m/z Expected	m/z Measured	Delta	Result	FWHM Expected	FWHM Measured	Delta	Result	Abundance
112.99	112.98	-0.01	Pass	1.20	1.11	-0.09	Pass	499008
302.00	301.92	-0.08	Pass	1.20	1.27	0.07	Pass	289965
601.98	601.92	-0.06	Pass	1.20	1.18	-0.02	Pass	854656
1033.99	1033.88	-0.11	Pass	1.20	1.21	0.01	Pass	1981569
1333.97	1333.83	-0.14	Pass	1.20	1.22	0.02	Pass	1923423

Analyzer: MS2 Polarity: Negative Width: Wide

m/z Expected	m/z Measured	Delta	Result	FWHM Expected	FWHM Measured	Delta	Result	Abundance
112.99	112.99	0.00	Pass	1.20	1.20	0.00	Pass	882855
302.00	301.97	-0.03	Pass	1.20	1.31	0.11	Pass	420831
601.98	602.04	0.06	Pass	1.20	1.32	0.12	Pass	996275
1033.99	1034.16	0.17	Pass	1.20	1.04	-0.16	Pass	1807598
1333.97	1334.13	0.16	Pass	1.20	0.93	-0.27	Pass	1944205
